# High serum ferritin alone as a predictor of mortality and hemophagocytic lymphohistiocytosis

**DOI:** 10.1002/jha2.153

**Published:** 2021-01-06

**Authors:** Shruti Kohli, Ritu Chadha, Neha Rastogi, Satya Prakash Yadav

**Affiliations:** ^1^ Pediatric Hematology Oncology and Bone Marrow Transplant Unit Medanta ‐ The Medicity Hospital Cancer Institute Gurgaon India; ^2^ Department of Hematopathology Medanta ‐ The Medicity Hospital Gurgaon India

**Keywords:** ferritin, hemophagocytic lympho histiocytosis, mortality

## Abstract

We report here utility of high serum ferritin alone as a predictor of mortality and diagnosis of hemophagocytic lymphohistiocytosis (HLH). We compared mortality in patients with high serum ferritin >5000 ng/mL versus <5000 ng/mL and looked for presence of HLH. Mortality was significantly higher (*P*‐value .0048) in patients with serum ferritin levels >5000 ug/dL. Of 21 patients with high serum ferritin, a median of three criteria were fulfilled to diagnose HLH. All patients had features of immune‐activation, and 76.2% patients had features of immune‐pathology favoring diagnosis of HLH. Serum ferritin can aid in prediction of mortality and help in the early diagnosis of HLH.

## 1 INTRODUCTION

Serum ferritin levels are high in hemophagocytic lymphohistiocytosis (HLH), and higher the ferritin values more likely is the diagnosis of HLH [1]. The clinical symptoms of HLH are common and similar to other infections, autoimmune conditions immunodeficiencies, and malignancies and therefore it may be difficult to clinch the diagnosis [2]. Timely diagnosis is crucial as suggested by more than 90% fatality rate in patients before the advent and use of immunomodulating drugs [1]. Ferritin is thought to play a role in the rapid detoxification of iron and facilitates iron nucleation, mineralization, and long‐term iron storage [3]. It is also a part of positive regulation of transcription in response to oxidative stress and proinflammatory cytokine signaling through nuclear factor kappa‐light‐chain‐enhancer of activated B cells (NF‐kB) pathway [4]. These functions of ferritin suggest that it might serve as a cytoprotective protein, minimizing oxygen free radical formation [3]. Small quantities of ferritin are also present in human serum and are elevated in conditions of iron overload and inflammation [5]. Measurement of ferritin levels does not require a specialized laboratory and is cheaper, and the report can be availed on the same day. Interleukin‐2 receptor alpha chain (IL‐2Ra) levels, on the other hand, requires a focused laboratory and is expensive and cumbersome to perform even though it is a sensitive test for diagnosis of HLH [6, 7]. In this retrospective study, we report here utility of high serum ferritin alone as a predictor of mortality and diagnosis of HLH.

## 2 METHODS

In this retrospective study, the Medanta hospital database was searched for all consecutive patients in whom serum ferritin levels had been done from January 2016 to December 2016. Patients with ferritin values above 500 ug/dL (as this has been used as a cutoff for diagnosis of HLH) were shortlisted. We looked for outcomes in terms of mortality in all these patients. Incidence of mortality was compared between patients with high serum ferritin levels >5000 ug/dL and those with levels <5000 ug/dL.

All patients with Ferritin levels > 5000 ng/mL were analyzed further for presence of HLH. Patients on regular packed red cell transfusions were excluded from the study. Medical records were retrieved, and further information supporting the diagnosis of HLH based on the HLH 2004 criteria [8] (fever, splenomegaly, other laboratory tests like liver function tests, triglyceride levels, and fibrinogen levels) in these patients was collected as recorded by treating physicians based on clinical signs/symptoms.

In many patients all the criteria could not be fulfilled. Therefore, an alternative definition to recognize HLH was required. Thus, patients were assessed on the basis of presence of features of immune activation (fever, hepatomegaly/splenomegaly, elevated ferritin, elevated CD25, elevated CD163) or abnormal immunopathology (cytopenias, decreased fibrinogen/increased triglycerides, hepatitis, hemophagocytosis, CNS involvement) as discussed by Jordan et al to describe the clinical patterns and pathogenesis of HLH [9]. Liver function tests were assessed in addition to these criteria as supportive evidence. Mean, median values, and *P*‐values were calculated wherever required.

## 3 RESULTS

### 3.1 Patients

During the study period there were a total of 128 patients with ferritin values >500 ng/mL, and 21 among these had ferritin levels >5000 ng/mL. The age range was 4‐82 years. The mean age was 39.7 years, and median was 38 years. Of the 21 patients, 14 were male and 7 females. Patients were from department of internal medicine (43%), rheumatology (14%), gastroenterology (10%), pediatric hemato‐oncology (14%), oncology (14%), and cardiology (5%).

### 3.2 Outcome

Mortality in patients with ferritin level >5000 ng/mL was 28.6% (6/21) versus 7.5% (8/107) in those with levels <5000 ng/mL (*P*‐value .0048).

### 3.3 ICU admissions

Ten of the 21 (47.6%) patients required ICU admission and prolonged hospital stay, and six out of 10 (60%) died.

### 3.4 HLH diagnostic criteria details

All patients with high serum ferritin (>5000 ng/mL) had fever (100%). Cytopenias (bi or pan) were present in 14 of 21 (66%) patients. Splenomegaly was present in six of 21 (28%) patients. Bone marrow aspiration showed presence of hemophagocytosis in three of five patients. Triglyceride levels were high in four of 12 patients. Hypofibrinogenemia was seen in one of six patients. Liver function tests had been done in all the patients. Deranged liver function (increase in enzyme level) was seen in 11 of 21 (52.3%) patients. Table 1 shows details of HLH criteria/alternative criteria and outcomes in patients with high serum ferritin >5000 ng/mL.

**TABLE 1:** HLH criteria/alternative criteria and outcome details in patients with high serum ferritin levels (>5000 ng/mL)

	Fever	Ferritin ng/dL	Hb g/dL	TLC /cumm	PLT /cumm	Bone marrow	Trig mg/dL	Fib mg/dL	Spleen	HLH score	LFT abnormal	Immune activation	Immune‐pathology	ICU	Outcome
1	Y	20000	6.6	40860	68000	ND	134	ND	No	3	Y	Y	Y	Y	Alive
2	Y	23700	12.4	1860	18000	ND	447	301	No	3	N	Y	Y	N	Alive
3	Y	21800	7.6	17180	442000	Y	169	156	No	3	Y	Y	Y	N	Alive
4	Y	14100	6.3	160	20000	ND	184	ND	N	3	Y	Y	Y	Y	Alive
5	Y	67500	8	67000	18000	ND	267	ND	N	4	N	Y	Y	Y	Alive
6	Y	7990	13	16850	534000	ND	397	ND	N	3	Y	Y	Y	N	Alive
7	Y	5210	13	15400	391000	ND	ND	ND	Y	3	N	Y	N	N	Alive
8	Y	6240	8.7	18050	40000	ND	ND	ND	N	3	N	Y	Y	Y	Alive
9	Y	8710	12.2	21450	308000	N	177	ND	Y	3	N	Y	N	N	Alive
10	Y	6380	7.9	1270	60000	ND	236	72	N	4	Y	Y	Y	Y	Died
11	Y	5200	10.8	12000	40000	ND	ND	ND	N	2	Y	Y	Y	N	Alive
12	Y	13500	10.8	14770	40000	N	ND	ND	Y	3	N	Y	N	Y	Died
13	Y	11600	7.7	3590	22000	Y	106	762	Y	5	Y	Y	Y	Y	Died
14	Y	5690	7.6	540	132000	Y	371	172	N	5	N	Y	Y	N	Alive
15	Y	18000	9	3290	80000	ND	198	ND	Y	3	Y	Y	Y	N	Alive
16	Y	25300	9.4	7320	40000	ND	ND	ND	Y	3	Y	Y	Y	Y	Died
17	Y	10700	7.5	8060	208000	ND	ND	ND	N	2	Y	Y	Y	N	Alive
18	Y	13000	6.1	59660	189000	ND	ND	ND	N	2	N	Y	N	Y	Died
19	Y	5380	16.1	11300	236000	ND	ND	ND	N	2	Y	Y	Y	N	Alive
20	Y	171000	8.9	9640	12000	ND	209	390	N	3	N	Y	Y	Y	Died
21	Y	5570	15.4	4680	130000	ND	ND	ND	N	2	N	Y	N	N	Alive

Abbreviations: Fib, fibrinogen; Hb, hemoglobin; HLH, hemophagocytic lympho histiocytosis; ICU, intensive care unit; LFT, liver function test; N, no; ND, not done; PLT, platelets; TLC, total leucocyte count; Trig, triglycerides; Y, yes.

To confirm diagnosis of HLH in 21 patients with high ferritin >5000 ng/dL, we could use only six of eight diagnostic criteria [8] as soluble IL2R and NK cell activity tests were not available at our center. Two patients had a score of 5 of 6. Bone marrow examination was performed for only five patients (due to lack of consideration of diagnosis of HLH). Therefore, we had essentially five criteria to work with, in the remaining 16 patients. Another two patients had a HLH score of 4 of 5. The average HLH score was 3.04, and the median was 3.

Features of immune activation were present in all 21 patients, and abnormal immune‐pathology was present in 16 of 21 (76.2%) patients. Deranged liver function was used as a supportive evidence of immunopathological involvement suggesting HLH in these patients. As per medical records none of the patients had documented central nervous system involvement, and no lumbar punctures were performed.

### 3.5 Treatment for HLH

Only nine of 21 (42.8%) patients received treatment mostly with steroids except one patient who received additional treatment with cyclosporine and etoposide.

## 4 DISCUSSION

Serum ferritin level has been included as a diagnostic criterion in HLH 2004 (>500 ng/mL). It is a simple and inexpensive test. In our patients only six criteria out of eight (as per HLH 2004 criteria [8]) were available to be tested as investigations like soluble CD25 and NK cell activity were not available. However, among the available tests also, not all were ordered due to lack of recognition of HLH as an entity. Even though all criteria were not met among these patients, there was a higher rate of ICU admissions and mortality in patients with serum ferritin levels above 5000 ng/mL compared to those with ferritin levels below 5000 ng/mL. Despite the fact that our patients had clinical symptoms of HLH, due to lack of data we needed an alternative system to help in the diagnosis. In a study by Jordan et al, it had been discussed the diagnosis of HLH can be challenging, and the key is to consider underlying immune mechanisms (immune activation and immune pathology) along with the other diagnostic HLH criteria [9]. Also they further suggested that in sick patients even if less than five criteria are met, along with evidence of deranged liver functions, the diagnosis of HLH should be considered, and further evaluation and treatment should be initiated early [9]. In our study, >50% of patients with high serum ferritin levels (>5000 ng/mL) had evidence of deranged liver functions, and 47% patients required ICU admission, suggesting a high morbidity. Our study suggests that HLH may be underdiagnosed due to inadequate evaluation as a result of lack of awareness, cost constraints, and delayed results. This leads to increased mortality as HLH is a potentially fatal if left untreated. In our study we looked at outcomes in terms of ICU admission and mortality.

It has been suggested previously that a serum ferritin value of >3000 ng/mL is of concern, and a value >10000 ng/mL is highly suggestive of HLH [1]. A previous study by Hearnshaw et al suggested that such high ferritin levels as seen in HLH are not observed in other illnesses [10]. We used a cutoff of >5000 ng/mL in our study since we observed that in our cohort the incidence of mortality was higher in these patients, and as we had a small cohort keeping a cutoff value >10000 ug/dL could probably lead to missed diagnosis.

In our study only nine patients had received treatment (steroids) of which only one patient received further appropriate treatment with etoposide and cyclosporine. Of the treated patients, two succumbed to death (probably due to delay in diagnosis and initiation of treatment). Another four patients died among 12 patients not treated for HLH. If a protocol had been in place to look for HLH in those with high ferritin at diagnosis then all 21 patients would have been properly worked up and treated and mortality could have been reduced.

Our study suggests that high serum ferritin is highly predictive of mortality and morbidity and correlates well with the diagnosis of HLH. It can be used as a first line investigation to facilitate early recognition of the risk of mortality and aid in timely diagnosis of HLH and appropriate treatment. Performing serum ferritin level is a trouble free, low cost intervention which can be easily done in all critically ill patients. High ferritin level (>5000 ng/mL) can be used as a cautionary test to warrant further HLH work up and expedite management even before other cumbersome investigations can be done.

## CONFLICT OF INTEREST

The authors declare that there is no conflict of interest that could be perceived as prejudicing the impartiality of the research reported.

## AUTHOR CONTRIBUTIONS

Study concept and design: Yadav. Drafting of the manuscript: Kohli and Yadav. Collection of data: Kohli and Chadha. Critical revision of the manuscript for important intellectual content: Rastogi.
